# *In vitro* Organic Acid Production and *In Vivo* Food Pathogen Suppression by Probiotic *S. thermophilus* and *L. bulgaricus*

**DOI:** 10.3389/fmicb.2019.00782

**Published:** 2019-04-17

**Authors:** Smith Etareri Evivie, Amro Abdelazez, Bailiang Li, Xin Bian, Wan Li, Jincheng Du, Guicheng Huo, Fei Liu

**Affiliations:** ^1^Key Laboratory of Dairy Science, Ministry of Education, College of Food Sciences, Northeast Agricultural University, Harbin, China; ^2^Food Science and Nutrition Unit, Department of Animal Science, Faculty of Agriculture, University of Benin, Benin City, Nigeria; ^3^Department of Dairy Microbiology, Animal Production Research Institute, Agriculture Research Center, Giza, Egypt; ^4^Department of Food Engineering, Harbin University of Commerce, Harbin, China

**Keywords:** *S. thermophilus*, *L. bulgaricus*, probiotics, foodborne pathogens, immunity, organic acid

## Abstract

Foodborne pathogens are a major source of morbidity and mortality worldwide. For this cause, exploring various effective ways of suppressing their spread is at the forefront of many research projects. The current study aims to investigate the *in vitro* organic acid production of *S. thermophilus* KLDS 3.1003 and *L. bulgaricus* KLDS 1.0207 strains, their *in vivo* suppression of and immuno-modulatory effects against *E. coli* ATCC 25922 and *S. aureus* ATCC 25923 pathogens. First, lactic and acetic acid production using three carbon sources – 1% glucose (control), 1% sucrose, and 1% fructo-oligosaccharides (FOS) – was determined by HPLC. For the *in vivo* section, a total of 40 BALB/c mice were purchased and divided into 10 treatment groups (control and nine treatments). Animals were given 1 week to acclimatize and then fed treatment diets for 14 days. Afterward, hematological (RBC, WBC, HB, PLT, Neutrophils, Eosinophils, Lymphocytes, and Monocytes) and histopathological analyses were carried out. All analyses were done in triplicate. Results show that lactic and acetic acid productions for both strains increased with supplementation and were highest after 1% FOS addition. Regardless of carbon source, *L. bulgaricus* KLDS 1.0207 produced higher (*P* < 0.05) amounts of lactic and acetic acids than *S. thermophilus* KLDS 3.1003. Also, generally better hematological parameters in probiotic groups than the control (*P* < 0.05) were observed. In some instances, mice in probiotic treatment groups had better immunity levels (lymphocytes, monocytes, neutrophils, eosinophils) than those in the control and pathogen groups. Histopathological studies showed that no anomalies were associated with *S. thermophilus* KLDS 3.1003 and *L. bulgaricus* KLDS 1.0207 administration. In conclusion, *S. thermophilus* KLDS 3.1003 and *L. bulgaricus* KLDS 1.0207 strains are not only probiotic candidates but can have therapeutic applications.

## Introduction

Nutritious foods sustain the complex requirements of the human organism, compensating the limitations of our digestive physiology and anabolic restrictions. Foods are also the perfect media for the growth of microorganisms rendering them inedible and even dangerous for consumption. It has been an everlasting struggle for humans to keep food safe and wholesome (Papadimitrou et al., 2015). Functional food acts as beneficial compounds or foods containing microorganisms exhibiting a pivotal role in strengthening and enriching health well-being and suppressing some strict disease, for instance, obesity, diabetes, atherosclerosis, heart disease, retinopathy, kidney toxicity, atherosclerosis, hypertension, diabetic foot ulcers, and cystic fibrosis (Ji et al., 2015; Abdelazez et al., 2017). Many lactic acid bacteria (LAB) strains serve as probiotics in functional foods.

Annually, it is estimated that as many as 30% of people in the industrialized countries suffer from foodborne illnesses (Ghanbari et al., 2013). Foodborne illnesses can spread in several forms from farm to fork so the importance of good management practices cannot be overemphasized (Hoffmann et al., 2017). The antagonistic effects of *L. helveticus* KLDS 1.8701 on some notable foodborne pathogens was recently reported but other promising LAB strains require more detailed studies (Bian et al., 2015). Next-generation sequencing (NGS) is one genomic tool that can be used to enhance the increased inhibition of notable foodborne pathogens by LAB (Didelot et al., 2012). Detailed studies of the various components, functionalities and potential uses of many beneficial LAB have been made possible by NGS (Lecomte et al., 2016; Landete, 2017).

The human intestinal ecosystem is one of the most densely populated and highly diverse microbial environment known to date, composed of stable and variable microbial groups. These microbes therein provide us with a significant metabolic potential encoded in the metagenome; thus the intestinal microbiota has been postulated as another organ of the human body (Sánchez et al., 2017). Thanks to NGS, it is now known that aberrant microbiota profiles have been found in highly prevalent diseases, such as inflammatory bowel diseases (IBD), irritable bowel syndrome (IBS) or colorectal cancer, among others (Gevers et al., 2014; Bullman et al., 2017; Bennet et al., 2018). Purported associations between gut microbiota composition and human health have resulted in the use of LAB as functional food ingredients (Arboleya et al., 2016). LAB colonization of the gut microbiota is believed to support appropriate immune development and prevent/limit the onset of certain gut diseases (Garcia-Mantrana et al., 2018). Many LABs can be used as probiotics to modulate the structure of the host gut microbiota, thus increasing their functionalities (Evivie et al., 2017; Li et al., 2017b).

Although a number of *in vitro* studies have been reported to show clearer correlation between immunity parameters and diet supplemented with some notable LAB strains (Evivie et al., 2017; Li et al., 2017b, 2018), more *in vivo* investigations are still required to validate these findings. Again, given the global deleterious impact of foodborne pathogens, a number of research initiatives are currently exploring ways of significantly lowering their effect which will improve healthy living. In the present study, two potential probiotic strains, *S. thermophilus* KLDS 3.1003 and *L. bulgaricus* KLDS 1.0207 were first evaluated for lactic and acetic acid production levels using different carbon sources *in vitro.* Afterward pathogens and probiotic bacteria were orally administered to 40 BALB/c mice in an *in vivo* trial where hematological and histological analyses were carried out. It is anticipated that the findings of this study would give further insights in the use of these strains as probiotics as well as show that immunity parameters of the study animals improved with LAB supplementation.

## Materials and Methods

### Bacteria Strains and Growth Conditions

*Streptococcus thermophilus* KLDS 3.1003 and *Lactobacillus bulgaricus* KLDS 1.0207 were obtained from the Key laboratory of Dairy Science (KLDS), Northeast Agricultural University, China. *E coli* ATCC 25922 *and S. aureus* ATCC 25923 were obtained from the Heilongjiang Entry-Exit Inspection and Quarantine Bureau, China. Pathogens were incubated as described by Bian et al. (2015). All chemicals and reagents used in this research were obtained from reliable suppliers in Harbin, China and of analytical grade.

### Effect of Carbon Sources on the Lactic and Acetic Acid Production of Bacterial Strains

This was carried out as described by Bian et al. (2015) with modifications. Briefly, glucose, sucrose, and fructo-oligosaccharide (FOS) (1% each) served as supplementary carbon sources to the M17 and mMRS broths with 1% glucose (control). Cell-free supernatants (CFS) were prepared as previously described. Lactic and acetic acid production of the different CFS samples were determined by High-Performance Liquid Chromatography (HPLC) as previously described by Zhang et al. (2011) with some modifications. Acid separation was achieved using an AMINEX HPX-87H ion exchange column (Bio-Rad Labs, Berkeley, CA, United States) and the organic acids were detected using a differential refraction detector with 5 mM H_2_SO_4_. Acid identification was carried out by comparing the retention times of the samples with that of the standards for organic acids. Experiments were carried out in triplicate.

### Animals

For the *in vivo* phase of this research, 40 female BALB/c mice (8 weeks old, weighing 15–25 g) were purchased from the Vital River Laboratory Animal Technology Company (Beijing, China). Feed and water were provided *ad libitum* in the first week of arrival. Afterward, 10 steel cages with eating and drinking sections were used to house the 10 treatment groups (4 mice/group). Each group was fed one diet throughout the study period of 14 days. The diets are as follows – Control (0.5% saline solution), T_ST_ (*S. thermophilus* KLDS 3.1003 only), T_LB_ (*L. bulgaricus* KLDS 1.0207 only), T_STLB_ (*S. thermophilus* + *L. bulgaricus*), T_EC_ (*E. coli* only), T_SA_ (*S. aureaus* only), T_STEC_ (*S. thermophilus* + *E. coli*), T_STSA_ (*S. thermophilus* + *S. aureus*), T_LBEC_ (*L. bulgaricus* + *E. coli*), and T_LBSA_ (*L. bulgaricus* + *S. aureus*). Feeding was done twice daily. The experiment was approved by the Institutional Animal Care and Use Committee of the Northeast Agricultural University under the approved protocol number Specific pathogen-free rodent management (SRM)-06.

### Hematological Analyses

After 14 days of feeding (excluding 1 week of acclimatization), all mice were fasted and sacrificed humanely. Blood samples were collected in heparinized tubes and assessed for red blood cell count (RBC), white blood cell count (WBC), hemoglobin (HB), platelet count (PLT), neutrophils, lymphocytes, monocytes, and eosinophils using the automatic hematological analyzer (Nihon Kohden, Tokyo, Japan).

### Gross Necropsy and Histopathological Studies

Liver, spleen, lung, and kidney were collected from each animal for gross necropsy analyses. Organs were stored in 10% neutral formalin for 18 h. Afterward, samples were immersed in paraffin, sliced into 5–10 μm thickness and stained with hematoxylin-eosin for examination using a light microscope (Available at the Center of Drug Safety Evaluation, Heilongjiang University of Chinese Medicine, Harbin).

### Statistical Analyses

All experiments were carried out at least in triplicates using independent assays. Data obtained from this study were analyzed by one-way analysis of variance (ANOVA) using the SPSS v22.0 software (SPSS Institute, United States) and values were expressed as Mean ± SD. Values of *P* < 0.05 were considered to be statistically significant.

## Results

### Lactic and Acetic Acid Production Profiles of Probiotic LABs

*S. thermophilus* KLDS 3.1003 and *L. bulgaricus* KLDS 1.0207 showed increased production of lactic and acetic acids when the M17 and MRS media were supplemented in the following increasing order: 1% glucose, 1% sucrose, and 1% FOS (Tables 1, 2). Acetic acid production was significantly (*P* < 0.05) lower than lactic acid production for both strains, though this improved slightly with supplemented glucose, sucrose, and FOS. S. *thermophilus* KLDS 3.1003 had its highest lactic acid production in the FOS-supplemented medium (23.31 ± 0.14 mg mL^-1^). This yield was significantly higher (*P* < 0.05) from that of the control (4.66 ± 0.09 mg mL^-1^) and 1% glucose medium (11.49 ± 0.22 mg mL^-1^). Lactic acid production between both strains was significantly different (*P* < 0.05). *S. thermophilus* KLDS 3.1003 also had its highest acetic acid production using the FOS-supplemented media (9.05 ± 0.06 mg mL^-1^). *L. bulgaricus* KLDS 1.0207 yielded more lactic (38.96 ± 0.06 mg mL^-1^) and acetic (11.77 ± 0.18 mg mL^-1^) acids than *S. thermophilus* KLDS 3.1003 and also had highest acid production levels after 1% FOS addition (*P* < 0.05). Genes for carbohydrate fermentation and additional genomic properties are also reported in this study (see Supplementary Figures S1, S2).

**Table 1:** Effect of carbon source on lactic acid production of *S. thermophilus* KLDS 3.1003 and *L. bulgaricus* KLDS 1.0207.

Lactic acid production (mg mL^-1^)				

LAB strains	Control	1% Glucose	1% Sucrose	1% FOS
*S. thermophilus* KLDS 3.1003	4.66 ± 0.09b	9.05 ± 0.06b	15.27 ± 0.20b	23.31 ± 0.14b
*L. bulgaricus* KLDS 1.0207	25.23 ± 0.33a	30.88 ± 0.17a	33.30 ± 0.25a	38.96 ± 0.06a


*All values (expressed as mean ± SD) were obtained from three independent experiments. Values with same alphabets along column are not significantly different (*P* > 0.05)*.

**Table 2:** Effect of carbon source on acetic acid production of *S. thermophilus* KLDS 3.1003 and *L. bulgaricus* KLDS 1.0207.

Acetic acid production (mg mL^-1^)				

LAB strains	Control	1% Glucose	1% Sucrose	1% FOS
*S. thermophilus* KLDS 3.1003	0.31 ± 0.02b	1.04 ± 0.11b	2.83 ± 0.03b	4.82 ± 0.07b
*L. bulgaricus* KLDS 1.0207	1.48 ± 0.02a	3.25 ± 0.10a	6.98 ± 0.09a	11.77 ± 0.17a


*All values (expressed as mean ± SD) were obtained from three independent experiments. Values with same alphabets along column are not significantly different (*P* > 0.05)*.

### Hematological Analyses

Hematological parameters of experimental animals in the control, T_ST_, T_STLB_, T_STEC_, and T_STSA_ groups are as shown in Table 3. For the WBC (10^3^ /μL), the T_STLB_, T_ST_, and C groups were 10.95, 10.77, and 10.80, respectively. The % lymphocytes in the above order were 74.83, 72.46, and 71.70, respectively. The general trend was that combined or single strain treatments gave superior performances compared to the Control. With the oral administration of pathogen alongside *S. thermophilus* KLDS 3.1003, there was no marked difference between the readings obtained. In RBC for example, the C, T_EC_, and T_STEC_ groups gave readings (expressed as ×10^3^/μL) of 6.85, 6.51, and 6.73, respectively. For the T_SA_ group, there readings were 6.85, 6.38, and 6.51, respectively. For the RBC, PLT, lymphocytes, monocytes, neutrophils and eosinophils parameters, T_STLB_-fed experimental animals had significantly (*P* < 0.05) higher values than both the T_ST_ and C groups. With the exception of HB, T_LBEC_ readings were generally better than T_EC_ (*P* < 0.05). Also, all T_LBSA_ values were significantly higher than T_SA_ group, suggesting that *L. bulgaricus* KLDS 1.0207 significantly inhibited *S. aureus in vivo* (Table 4).

**Table 3:** Hematological parameters of study animals after oral administration of *S. thermophilus* KLDS 3.1003 for 14 days.

Treatments							

Parameters	Control	T_ST_	T_STLB_	T_EC_	T_SA_	T_STEC_	T_STSA_
RBC (×10^6^ /μL)	6.85 ± 0.08b	6.90 ± 0.04b	7.06 ± 0.05a	6.51 ± 0.04d	6.38 ± 0.05e	6.73 ± 0.05c	6.51 ± 0.06d
WBC (×10^3^ /μL)	10.80 ± 0.10a	10.77 ± 0.12b	10.95 ± 0.06a	10.81 ± 0.09a	10.44 ± 0.06c	10.73 ± 0.04b	10.51 ± 0.10c
HB (g/L)	138.29 ± 0.57a	138.46 ± 0.49a	139.70 ± 0.53a	136.58 ± 0.47c	136.03 ± 0.21c	137.31 ± 0.31b	137.97 ± 0.16b
PLT (×10^3^ /μL)	1215.57 ± 6.32b	1215.48 ± 3.03b	1231.87 ± 4.68a	1200.89 ± 0.86d	1199.15 ± 0.74d	1209.14 ± 1.85c	1200.69 ± 0.54d
Neutrophils (%)	23.05 ± 0.42b	23.79 ± 0.23a	23.79 ± 0.20a	22.49 ± 0.09c	22.34 ± 0.13c	23.19 ± 0.22b	22.80 ± 0.05c
Lymphocytes (%)	71.70 ± 0.97b	72.46 ± 0.32b	74.83 ± 0.27a	70.39 ± 0.59	70.10 ± 0.10c	71.33 ± 0.32c	71.28 ± 0.29c
Monocytes (%)	5.11 ± 0.18b	5.34 ± 0.03b	6.05 ± 0.08a	4.75 ± 0.06c	4.69 ± 0.03c	4.98 ± 0.06b	4.77 ± 0.06c
Eosinophils (%)	1.27 ± 0.07b	1.31 ± 0.02b	1.45 ± 0.07a	1.19 ± 0.01c	1.18 ± 0.02d	1.22 ± 0.02c	1.19 ± 0.01c


*Values are expressed as Mean ± SD, Control, sterile normal saline; RBC, red blood cell count; WBC, white blood cell count; HB, hemoglobin; PLT, platelet count. Values with the same alphabet along the same row are not significantly different (*P* > 0.05)*.

**Table 4:** Hematological Parameters of Study animals after oral administration of *L. bulgaricus* KLDS 1.0207 for 14 days.

Treatments							
Parameters	Control	T_LB_	T_STLB_	T_EC_	T_SA_	T_LBEC_	T_LBSA_
RBC (×10^6^ /μL)	6.85 ± 0.08b	6.99 ± 0.08a	7.06 ± 0.05a	6.51 ± 0.04e	6.38 ± 0.05e	6.76 ± 0.07c	6.69 ± 0.09d
WBC (×10^3^ /μL)	10.80 ± 0.10a	10.97 ± 0.07a	10.95 ± 0.06a	10.81 ± 0.09a	10.44 ± 0.06c	10.85 ± 0.08a	10.65 ± 0.03b
HB (g/L)	138.29 ± 0.57a	139.95 ± 0.60a	139.70 ± 0.53a	136.58 ± 0.47c	136.03 ± 0.21c	136.37 ± 0.38b	137.45 ± 0.47b
PLT (×10^3^ /μL)	1215.57 ± 6.32b	1238.14 ± 5.88a	1231.87 ± 4.68a	1200.89 ± 0.86c	1199.15 ± 0.74d	1205.52 ± 0.68c	1205.39 ± 1.50c
Neutrophils (%)	23.05 ± 0.42b	23.60 ± 0.51b	23.79 ± 0.20b	22.49 ± 0.09c	22.34 ± 0.13c	23.12 ± 0.13b	24.38 ± 0.40a
Lymphocytes (%)	71.70 ± 0.97b	73.68 ± 0.86a	74.83 ± 0.27a	70.39 ± 0.59c	70.10 ± 0.10c	71.35 ± 0.40b	73.11 ± 0.10a
Monocytes (%)	5.11 ± 0.18b	6.27 ± 0.16a	6.05 ± 0.08a	4.75 ± 0.06c	4.69 ± 0.03c	5.59 ± 0.08b	4.90 ± 0.04c
Eosinophils (%)	1.27 ± 0.07b	1.44 ± 0.05a	1.45 ± 0.07a	1.19 ± 0.01c	1.18 ± 0.02d	1.29 ± 0.02b	1.21 ± 0.01c


*Values are expressed as Mean ± SD, Control, sterile normal saline; RBC, red blood cell count; WBC, white blood cell count; HB, hemoglobin; PLT, platelet count. Values with the same alphabet along the same row are not significantly different (*P* > 0.05)*.

### Histopathology and Necropsy Analyses

**FIGURE 1:**
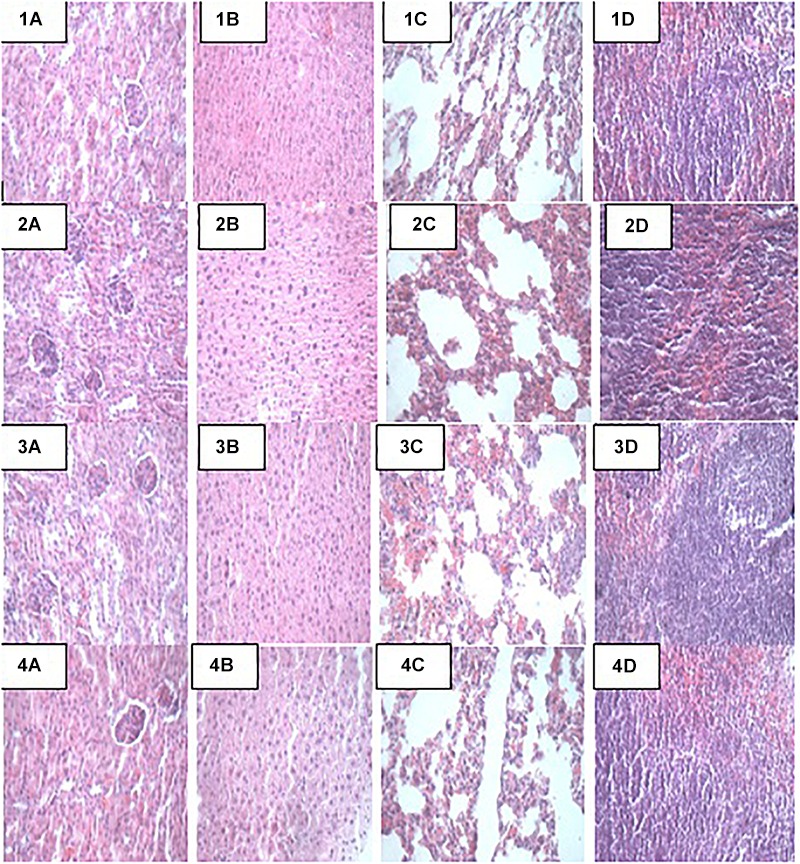
Representative photomicrographs of organs of mice fed Control, T_ST_, T_LB_, and T_STLB_ diets **1A–1D**: Control (Kidney, liver, lung, and spleen, respectively), **2A–2D**: T_ST_ (Kidney, liver, lung, and spleen, respectively), **3A–3D**: T_LB_ (Kidney, liver, lung, and spleen, respectively), and **4A–4D**: T_STLB_ (Kidney, liver, lung, and spleen, respectively).

**FIGURE 2:**
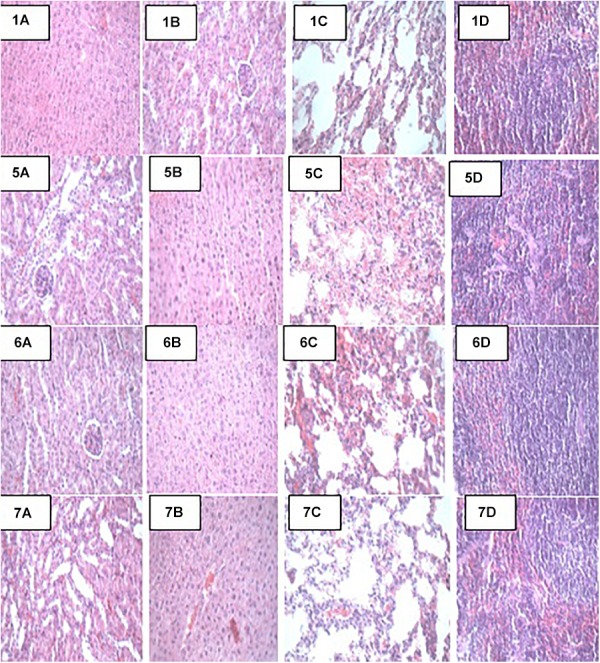
Representative photomicrographs of organs of mice fed Control, T_SA_, T_STSA_, and T_LBSA_ diets **1A–1D**: Control (Kidney, liver, lung, and spleen, respectively), **5A–5D**: T_SA_ (Kidney, liver, lung, and spleen, respectively), **6A–6D**: T_STSA_ (Kidney, liver, lung, and spleen, respectively), and **7A–7D**: T_LBSA_ (Kidney, liver, lung, and spleen, respectively).

**FIGURE 3:**
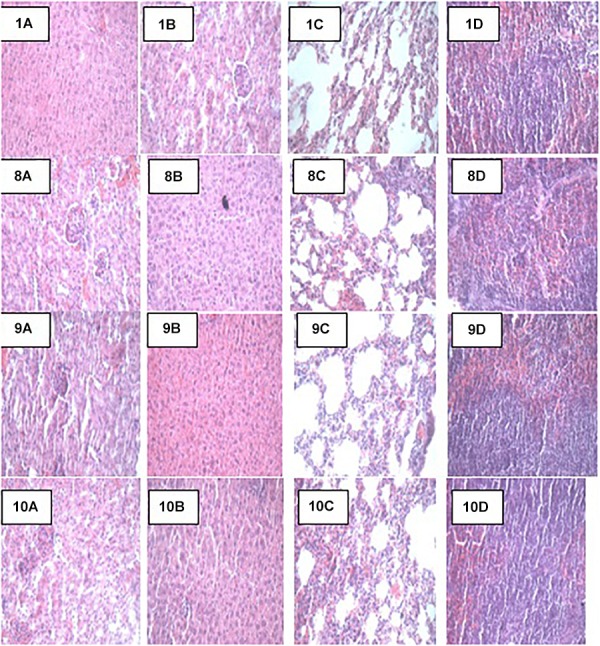
Representative photomicrographs of organs of mice fed Control, T_EC_, T_STEC_ and T_LBEC_ diets **1A–1D**: Control (Kidney, liver, lung, and spleen, respectively), **8A–8D**: T_EC_ (Kidney, liver, lung, and spleen, respectively), **9A–9D**: T_STEC_ (Kidney, liver, lung, and spleen, respectively), and **10A–10D**: T_LBEC_ (Kidney, liver, lung, and spleen, respectively).

At the end of the 14-day *in vivo* study, gross necropsy and histopathological examination of BALB/c mice were carried out. Results show that no organ damages were detected in mice of all the groups (Figure 1–3). Although the pathogen and LAB-pathogen groups showed no signs of damage, we recorded some mortality at the 7th and 14th day of this study. From the micrographs, no histopathological abnormalities were related to *S. thermophilus* KLDS 3.1003 and *L. bulgaricus* KLDS 1.0207 administration were observed.

## Discussion

### Supplementary Carbon Sources on Lactic and Acetic Acid Production

Organic acids are important pathogen-inhibiting compounds secreted by LAB (Vieco-Saiz et al., 2019). Results from this study showed that *L. bulgaricus* KLDS 1.0207 produced significantly higher amounts of lactic and acetic acids than *S. thermophilus* KLDS 3.1003. Although we had recently investigated the organic acid production of *L. helveticus* KLDS 1.8701 based on different carbon sources used, specific organic acids were not elucidated (Bian et al., 2015). The present study not only investigates more strains but also specific organic acid production levels (lactic and acetic). In a quantitative HPLC study by Gezginc et al. (2015), much higher yields of lactic acid by *S. thermophilus* (0–77.9 mg kg^-1^) and *L. bulgaricus* (0–103.5 mg kg^-1^) strains isolated from Turkish yogurts were obtained, both studies agree that organic acid production are both strain and species-specific. This is further corroborated by a recent study showing the production of six different organic acids by LAB using fish infusion broth (Özcelik et al., 2016). Moreover, the present study reports significant increase in the lactic and acetic acid yields in the following order: 1% FOS > 1% sucrose > 1% glucose, suggesting that the nature of the growth medium plays a critical role in organic acid production by probiotics. FOS has been shown to not only enhance the antagonistic potentials of LAB strains but is also a more efficient carbon source than glucose (Tejero-Sariñena et al., 2012; Bian et al., 2015; Kim et al., 2019). Furthermore as a prebiotic, it has been shown to stimulate the growth of probiotic LABs and thus increase their antagonistic effects on pathogenic organisms, primarily by enhancing the production of organic acids (lactic, acetic, propionic, and butyric) as well as a significant decrease in the pH of the fermented broth (Yang et al., 2011; Tejero-Sariñena et al., 2012; He et al., 2016). The development of effective synbiotics based on these findings thus merits further studies as this is more synergistic that individual effects of probiotics and prebiotics (Evivie, 2013; Evivie et al., 2017).

### Effects of Probiotic Administration on Hematological Parameters

Dietary components play a critical role either in the spread or treatment of a number of health disorders. As such, they remain a key area of research interest (Xu et al., 2015; Al-muzafar and Amin, 2017). In addition, *in vivo* animal studies are important because the body functioning of animals is in many instances, similar to that of humans (Shokryazdan et al., 2016). Although the potential antibiotic and probiotic properties of these and other selected LAB strains have been earlier reported, the present study further assessed their pathogen-inhibiting roles *in vivo*. Both *S. thermophilus* KLDS 3.1003 and *L bulgaricus* KLDS 1.0207 significantly suppressed the pathogenicity of *E. coli* ATCC 25922 and *S. aureus* ATCC 25923. Also, there was no significant difference between the control and treatment (T_LB_, T_STLB_, T_STEC_, T_LBEC_, T_STSA_, and T_LBSA_) groups. The parameters assessed are also used to measure the standard of immunity, therefore this study compared the immune-responses of animals in the control group with those in the various treatment groups. The neutrophils, lymphocytes, monocytes, and eosinophil levels in the T_ST_ group were 23.60, 73.68, 6.27, and 1.44, respectively. For the same parameters, the C values were 23.05, 71.70, 5.11, and 1.27, respectively. Interestingly, even in the T_ST_, T_STEC_, T_LBEC_, T_STSA_, and T_LBSA_ groups, the standard of immunity were still higher than the control group. These imply that single and co-culture doses of *S. thermophilus* KLDS 3.1003 and *L. bulgaricus* KLDS 1.0207 can potentially improve immunity levels in humans. It has also been recently shown that *L. helveticus* KLDS 1.8701 can raise immunity standards following pathogenic *E. coli* 0157:H7 infection in mice (Bian et al., 2016). No significant differences (*P* > 0.05) between the control and experimental groups were observed in the present study. Furthermore, food containing probiotics improves IgA and other immunity parameters (Aboderin and Oyetayo, 2006; Shokryazdan et al., 2016). Niamah et al. (2017) also confirmed not only improvements in hematological criteria of experimental mice but also in the standard of immunity. These recent *in vivo* findings as well as the present study confirm that beneficial bacteria strains can be potential ingredients in food formulations in view of improving health and wellbeing. From the T_STLB_ results obtained, it is suggestive that a co-culture of *S. thermophilus* KLDS 3.1003 and *L. bulgaricus* KLDS 1.0207 may be more effective than individual strains. In treating metabolic syndrome (MS) in high-fat diet (HFD)-fed rats, Wang et al. (2015) postulated that a combination of individual strains may be a more effective treatment than single-strain doses. Recent findings showing that yogurt cultures of *S. thermophilus* and *L. bulgaricus* can modulate immune responses to improve some gut microbiota dysfunctions, are also in consonance with the present study (Usui et al., 2019; Wasilewska et al., 2019). Further studies of this co-culture in suppressing the growth of other pathogens are thus warranted. The general trend of values obtained were in consonance with our previous findings that *S. thermophilus* KLDS 3.1003 was less effective against *S. aureus* than *E. coli*. RBC, WBC, HB, and PLT values for the T_LB_*-*fed animals were 6.99, 10.97, 139.95, and 1238.14, respectively. These values were far better than both T_ST_ and C. T_LBEC_ and T_LBSA_-fed animals generally had superior performance to T_STEC_ and T_STSA_-fed animals in terms of hematological results. These may be attributed to strain differences and specificity as a preliminary *in vitro* trial showed that that the CFS of *L. bulgaricus* KLDS 1.0207 had more antimicrobial effects against *S. aureus* ATCC 25923 and *E. coli* ATCC 25922 than *S. thermophilus* KLDS 3.1003 (data not shown).

### Effects of Probiotic Administration on Histopathological Analyses

Apart from their immune-modulatory effects, it has been suggested that probiotics have an effect on the gut microbiome by their antimicrobial activities directed toward intestinal pathogens (Rioux et al., 2005). Results from this study show that there were no signs of organs damage in all study animals. Histopathology can provide clear clinical advantage, *S. thermophilus* KLDS 3.1003 and/or *L. bulgaricus* KLDS 1.0207 intake did not result in any histologic abnormalities. This, to our knowledge, is the first study elucidating the histopathological evidence of non-inflammation of these two LAB strains. The oral administration of low (1 × 10^9^ CFU/kg BW) and high (1 × 10^10^ CFU/kg BW) doses of *L helveticus* KLDS 1.8701 did not give histological or clinical signs suggestive of organ damage (Li et al., 2017a). Probiotic therapy is becoming increasingly popular in veterinary medicine, and has been recommended for the treatment or prevention of a variety of gastrointestinal disorders. However, few objective studies attesting clinical efficacy of probiotics are available (Chang et al., 2018). In a recent study using dogs with idiopathic IBD, Rossi et al. (2014) reported that probiotics treatment (VSL#3) lowered histopathological scores compared to a combined treatment of prednisone and metronidazole. These researchers also noted that the probiotic-treated specifically conferred protection associated with an enhancement of regulatory T-cell markers (FoxP3+ and TGF-b+) and not in animals receiving combination therapy. Also, Al-muzafar and Amin (2017) showed that probiotics not only ameliorated the effects of high-fat sucrose diet (HFSD) in male albino rats, but the HFSD + probiotics group also showed improved lipid profiles, better leptin and resistin levels, and better TNF-α and IL-6 levels than the HFSD-only group. There were no histopathological signs of non-alcoholic fatty liver disease (NAFLD) in the HFSD + probiotic group which is in consonance with the findings of the present study.

## Conclusion

Foodborne pathogens are significantly deleterious to normal body functioning and researchers are currently investigating novel and effective ways mitigating their impact. The *in vitro* and *in vivo* suppression of *S. aureus* ATCC 25923 and *E. coli* ATCC 25922 by *S. thermophilus* KLDS 3.1003 and *L. bulgaricus* KLDS 1.0207 were investigated in this report. Lactic and acetic acid productions increased with supplementation with *L. bulgaricus* KLDS 1.0207 being significantly higher than *S. thermophilus* KLDS 3.1003 (*P* < 0.05). Hematological and serum biochemical parameters generally improved in animals fed LAB-treated diets, and in some instances, T_STLB_-fed animals fared better than T_ST_ and T_LB_-fed ones. No histological damages were observed in the organs in the control or treatment groups. For all *in vitro* and most of the *in vivo* parameters studied, results for *L. bulgaricus* KLDS 1.0207 were better than *S. thermophilus* KLDS 3.1003. In all, both strains can be adjudged good probiotic candidates and can have further therapeutic applications.

## Data Availability

The datasets generated for this study can be found in National Center for Biotechnology Information, CP016877.

## Ethics Statement

This study was carried out in accordance with the recommendations of “the Institutional Animal Use and Care Committee of the Northeast Agricultural University.” The protocol was approved by the above-mentioned committee “under the approved protocol number Specific pathogen-free rodent management (SRM)-06.”

## Author Contributions

SE and GH conceptualized the study. SE and AA developed the methodology. SE, AA, BL, and XB carried out the experiments. WL, JD, and FL provided technical support. SE, AA, and BL collected and analyzed the data. GH supervised the study. SE drafted the manuscript. All the authors revised and approved the final manuscript draft, and contributed equally to the publishing of this manuscript.

## Conflict of Interest Statement

The authors declare that the research was conducted in the absence of any commercial or financial relationships that could be construed as a potential conflict of interest.
