# Supplementary material for: In vitro Organic Acid Production and In Vivo Food Pathogen Suppression by Probiotic S. thermophilus and L. bulgaricus
Source: Front Microbiol. 2019 Apr 17;10:782. doi: 10.3389/fmicb.2019.00782 (PMC6479190; doi:10.3389/fmicb.2019.00782)
Supplement: Supplementary file 1 [file Data_Sheet_1.docx]

Supplementary Material

***In vitro o*rganic acid production and *in vivo* food pathogen suppression by probiotic *S. thermophilus* KLDS 3.1003 and *L. bulgaricus* KLDS 1.0207**

Smith Etareri Evivie^a,b, †^, Amro Abdelazez^a,c^, Bailiang Li^a, †^, Xin Bian^d^, Wan, Li^a^, Jincheng Du^a^, Guicheng Huo^a^* & Fei Liu^a^

^1^Key Laboratory of Dairy Science, Ministry of Education, College of Food Sciences, Northeast Agricultural University, Harbin 150030, PR China

^2^ Food Science and Nutrition Unit, Department of Animal Science, Faculty of Agriculture, University of Benin, PMB 1154 Benin City, Nigeria\

^3^ Department of Dairy Microbiology, Animal Production Research Institute, Agricultural Resear ch Centre, Dokki, Giza, 12618, Egypt

^4^ Department of Food Engineering, Harbin University of Commerce, Harbin 150028, PR China

^†^These authors contributed equally to this work.

*** Correspondence:** Professor Guicheng Huo, Key Laboratory of Dairy Science, Northeast Agricultural University, College of Food Sciences, 59 Mucai Street, Harbin, 150030, China. gchuo@neau.edu.cn, Tel: 0086-451- 55191807, Fax: 0086-451-55190577.

Supplementary Figures

**
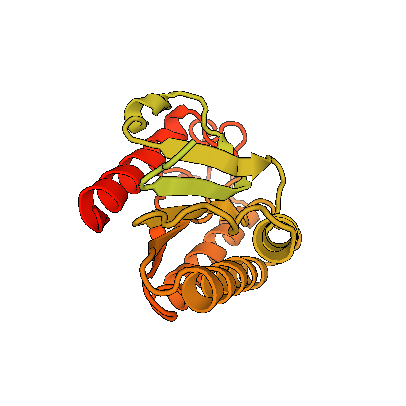

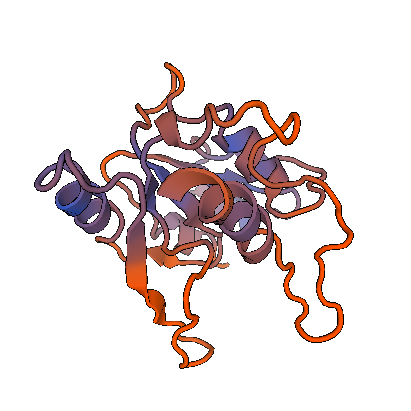
**

**B**

**A**

**
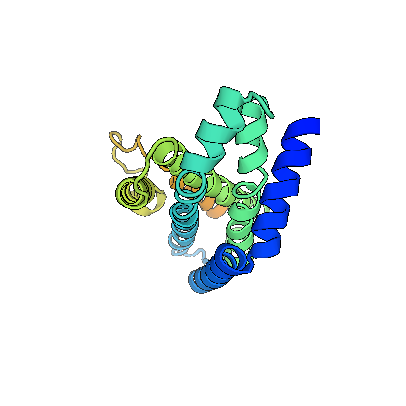

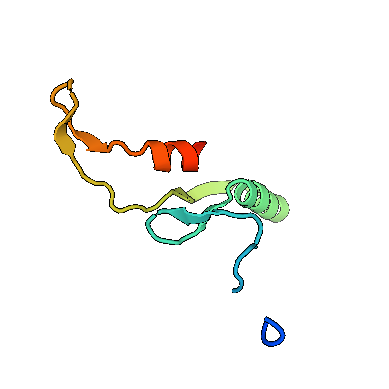
**

D

**C**


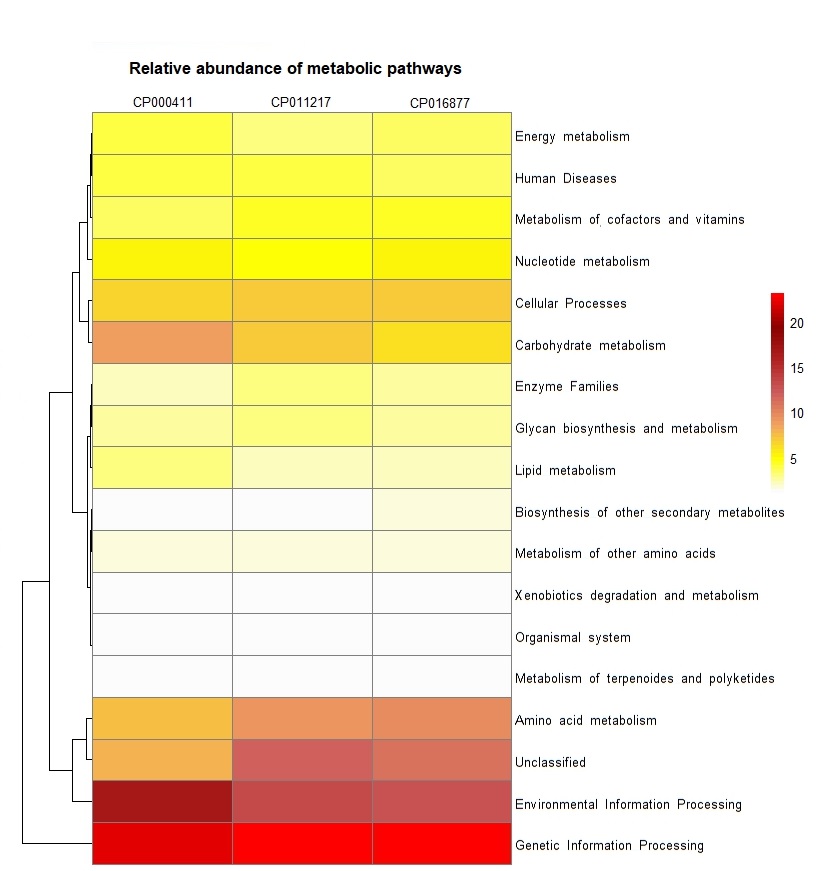


## Supplementary Figures

S1Three dimensional structures of 4 key enzymes involved in carbohydrate fermentation in *S. thermophilus* KLDS 3.1003 (A) Acetyl transferase (B) Rhamnosyl trnnsferase (C) glycosyl transferase, and (D) 6-phospho-beta glucosidase

S2 A heatmap comparing the relative abundance of metabolic pathways of three *S. thermophilus* strains – LMD-9 (CP00041), SMQ-301 (CP011217) and KLDS 3.1003 (CP016877). KLDS 3.1003 is distinct in that it alone has metabolic pathways for the biosynthesis of other secondary metabolites as well as having the highest amino acid metabolism pathways.

**
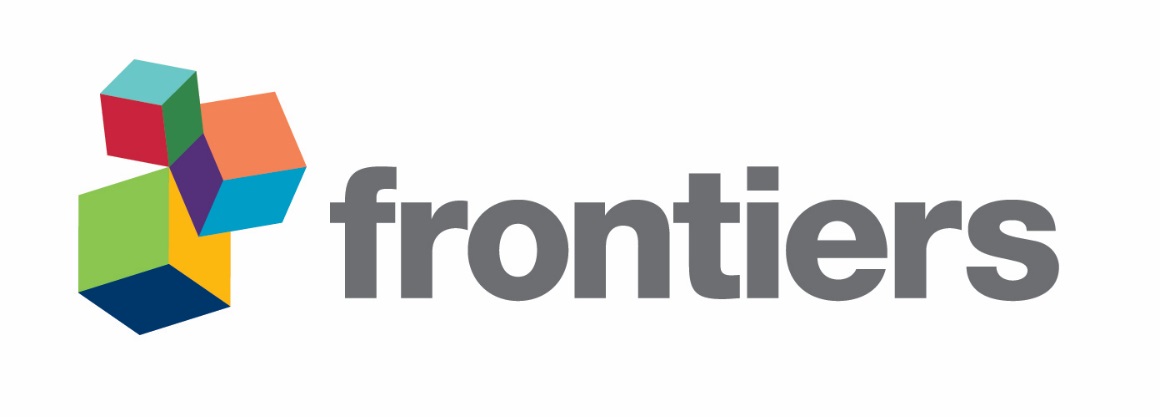
**

**Supplementary Figure 1.** The figure legends are required to have the same font as the main text, 12 point normal Times New Roman, single spaced. Please use a single paragraph for each legend and prepare the figures keeping in mind the PDF layout.
